# Supplementary material for: The interactome of intact mitochondria by cross-linking mass spectrometry provides evidence for coexisting respiratory supercomplexes
Source: Mol Cell Proteomics. 2017 Dec 8;17(2):216–32. doi: 10.1074/mcp.RA117.000470 (PMC5795388; doi:10.1074/mcp.RA117.000470)
Supplement: Supplemental Data [file supp_RA117.000470_133922_0_supp_23978_fzffwf.zip › spectra_annotation/mito_DR_spectra_annotation/236-1-8-1-8-1.pdf]

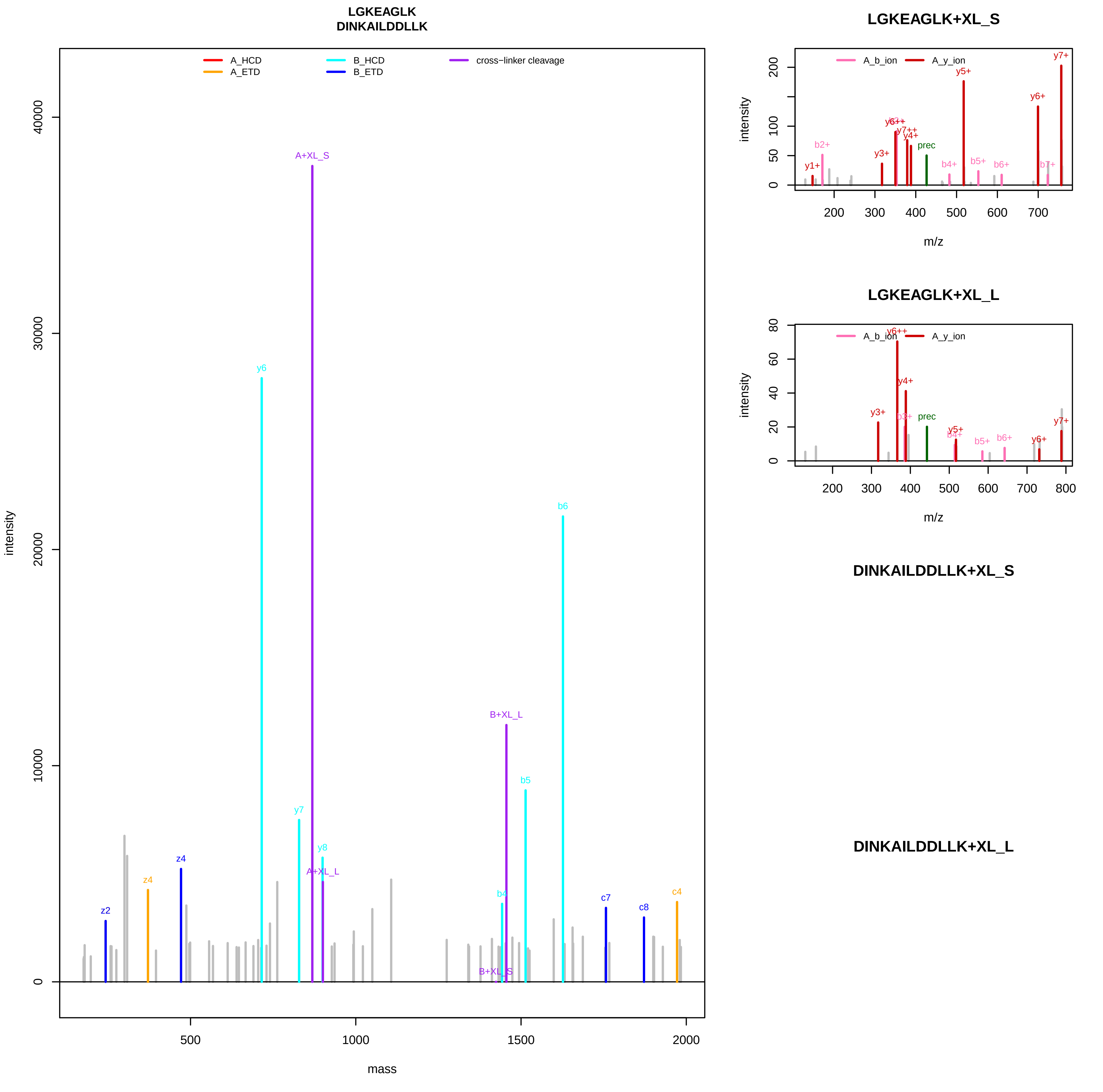
LGKEAGLK
DINKAILDDLLK
A_HCD
A_ETD
B_HCD
B_ETD
cross−linker cleavage
intensity
mass
0
10000
20000
30000
40000
500
1000
1500
2000
z2
z4
y6
y7
y8
A+XL_S
A+XL_L
B+XL_L
B+XL_S
b4
b5
b6
c7
c8
c4
LGKEAGLK+XL_S
A_b_ion
A_y_ion
intensity
m/z
y1+
b2+
y3+
y5+
y6+
y7+
y4+
prec
b4+
b5+
b6+
LGKEAGLK+XL_L
A_b_ion
A_y_ion
intensity
m/z
y6++
y4+
y3+
y5+
b5+
b6+
y6+
y7+
DINKAILDDLLK+XL_S
DINKAILDDLLK+XL_L
